# A Comparative Study on Fetal Heart Rates Estimated from Fetal Phonography and Cardiotocography

**DOI:** 10.3389/fphys.2017.00764

**Published:** 2017-10-17

**Authors:** Emad A. Ibrahim, Shamsa Al Awar, Zuhur H. Balayah, Leontios J. Hadjileontiadis, Ahsan H. Khandoker

**Affiliations:** ^1^Department of Electrical and Computer Engineering, Khalifa University of Science and Technology, Abu Dhabi, United Arab Emirates; ^2^Department of Obstetrics and Gynaecology, College of Medicine and Health Science, UAE University, Al Ain, United Arab Emirates; ^3^Department of Electrical and Computer Engineering, Aristotle University of Thessaloniki, Thessaloniki, Greece; ^4^Department of Biomedical Engineering, Khalifa University of Science and Technology, Abu Dhabi, United Arab Emirates; ^5^Department of Electrical and Electronic Engineering, University of Melbourne, Parkville, VIC, Australia

**Keywords:** fetal heart sounds (fHS), phonocardiography (PCG), phonograms, cardiotocography (CTG), blind source separation (BSS), vibration sensors

## Abstract

The aim of this study is to investigate that fetal heart rates (fHR) extracted from fetal phonocardiography (fPCG) could convey similar information of fHR from cardiotocography (CTG). Four-channel fPCG sensors made of low cost (<$1) ceramic piezo vibration sensor within 3D-printed casings were used to collect abdominal phonogram signals from 20 pregnant mothers (>34 weeks of gestation). A novel multi-lag covariance matrix-based eigenvalue decomposition technique was used to separate maternal breathing, fetal heart sounds (fHS) and maternal heart sounds (mHS) from abdominal phonogram signals. Prior to the fHR estimation, the fPCG signals were denoised using a multi-resolution wavelet-based filter. The proposed source separation technique was first tested in separating sources from synthetically mixed signals and then on raw abdominal phonogram signals. fHR signals extracted from fPCG signals were validated using simultaneous recorded CTG-based fHR recordings.The experimental results have shown that the fHR derived from the acquired fPCG can be used to detect periods of acceleration and deceleration, which are critical indication of the fetus' well-being. Moreover, a comparative analysis demonstrated that fHRs from CTG and fPCG signals were in good agreement (Bland Altman plot has mean = −0.21 BPM and ±2 *SD* = ±3) with statistical significance (*p* < 0.001 and Spearman correlation coefficient ρ = 0.95). The study findings show that fHR estimated from fPCG could be a reliable substitute for fHR from the CTG, opening up the possibility of a low cost monitoring tool for fetal well-being.

## 1. Introduction

Fetal well-being monitoring in a non-invasive way is a crucial step for obsterician and midwives to understand the fetal health status. In this endeavor, various non-invasive monitoring approaches have been adopted. Fetal echocardiography (fECHO) based on ultrasound signals (Nassit and Berbia, 2015b) is very widely used approach for monitoring fetal well-being. fECHO uses sound waves that “echo off” of the structures of the fetus' heart to produce a picture, or echocardiogram, of the fetus heart's interior, and it is mainly used for the diagnosis of congenital heart defects (20th–23rd week of pregnancy). Another method used for the fHR monitoring is fetal magnetocardiography (fMCG), which is a recording of the magnetic field of the fetal heart, using SQUID sensors placed over maternal abdomen (Peters et al., 2001). It, however, allows for easy fHR morphological analysis, due to its high signal-to-noise ratio (SNR) ouput. Nevertheless, fMCG is expensive and needs trained staff. Non-invasive fetal electrocardiography (fECG) is one of them which is based on the maternal abominal Electrocardiogram (abECG) (Freeman et al., 1997). In fECG, the fetal heart rate (fHR) is monitored through the acquired abECG by placing electrodes on the mother's abdomen and extracting fHR with the application of signal processing techniques on the abECG (Hyvarinen and Oja, 2001; Kanjilal and Saha, 2012; Wu et al., 2013; Nassit and Berbia, 2015a). A routine clinical method is the Cardiotocography (CTG), which is used during pregnancy to monitor both the fetal heart and the contractions of the uterus (Grivell et al., 2015). CTG involves the placement of two transducers onto the abdomen of a pregnant women; one transducer records the fHR using ultrasound, whereas the other transducer monitors the contractions of the uterus, by measuring the tension of the maternal abdominal wall, providing an indirect indication of intrauterine pressure. The current CTG technology is well-advanced and it is a routine part of modern obstetrics in all developed countries. One of the disadvantages, however, of CTG is its high sensitivity to different types of noise generated by maternal movements, requiring frequent repositioning of the ultrasound transducers. Moreover, due to the potential harmful effects of sustained ultrasonic radiation on the fetus (not well-understood so far), CTG seems not suitable for long-term continuous fetal monitoring. Moreover, CTG does not provide any information about beat-to-beat variability (Nageotte, 2015).

An alternative, cheaper and non-invasive fHR extraction can be done by fetal phonocardiography (fPCG). Its primitive form has its roots in the qualitative auscultation of fHS by general practitioners (dated back to the 1750s as a discovery by Kergardec, Marsac, and Kennedy; Sartwelle, 2012) via the so-called Pinard's stethoscope. The basic principle behind the fPCG is that the heart's mechanical activity is accompanied by the generation of a variety of characteristic sounds. These sounds are associated with changes in the speed of blood flow, as well as with the opening and closing of heart valves and could provide diagnostic information, accordingly (Tang et al., 2016). In modern fPCG, fHS are picked through sound transducers placed on the mother's abdomen and different characteristics, such as rate, frequency, and duration or changes in individual parts of the recorded cardiac acoustic signal can be measured. Apart from sound transducers, fiber-optic sensors have recently been introduced for the fPCG recordings (Martinek et al., 2016).

One of the main challenges faced in fHR estimation from fPCG analysis is the difficulty in extracting information from very noisy transducer data because data are affected by acoustic damping, manly due to amniotic fluid and digestive activity in addition to four main sources of sounds such as fetal and maternal heart contractions, maternal breathing and fetal movements. Other secondary sources of noise include shear noises due to transducers movement. All these noises embedded in the fPCG signal in time- and frequency-domain make the extraction of fHR very challenging. fPCG signals from the sound transducers are of low energy; hence, the SNR is quite low.

To address the aforementioned problem, various signal processing tools and techniques have been proposed and applied in the extraction of valid information from fPCG recordings. Initially FIR/IIR Filtering (Ginsburg et al., 1964; Talbert et al., 1986; Adithya et al., 2017) was used, which has low computational complexity yet high chance of failure to separate the desired fPCG components. FIR/IIR Filtering is suitable for pre-conditioning, e.g., 50 Hz notch filter. Some heuristic methods such as spectral substraction (Kovacs et al., 2006; Ruffo et al., 2010; Adithya et al., 2017) provide some noise enhancement via the artifact attenuation implemented at low computational complexity, yet they are mainly useful in post processing for fPCG classification. Also, spectral subtraction (Chen et al., 2006; Adithya et al., 2017) works under stationary noise model and its performance is linked to the quality of noise estimation. Adaptive filtering (Goovaerts et al., 1991; Adithya et al., 2017) showed poor performance in fPCG extraction. This could be improved if the maternal heart sounds (mHS) were first estimated and canceled using a multi-channel system. Kalman filtering (Adithya et al., 2017) needs accurate reference signal, as it has high computational complexity. Wavelet Transform (Kosa et al., 2008; Kovacs et al., 2011; Fodor et al., 2012; Adithya et al., 2017; Koutsiana et al., 2017), showed good performance in de-nosing noisy fPCG and analyzing the fetal heart sounds (fHS), but it lacks the ability to finely de-noise the overlapped frequency components. Independent Component Analysis (ICA) (Nigam and Priemer, 2004; Jimenez-Gonzalez and James, 2008; Jiménez-Gonzàlez and James, 2010; Jimenez and James, 2013) assumes source components as independent (which is hardly met in case of abdominal sources of sounds) and needs post processing with high computational complexity. Empirical Mode Decomposition has also been applied in the fHR extraction from simulated fPCG, yet it still needs evaluation in the context of real fPCG data (Taralunga et al., 2015). Moreover, many algorithms, such as, Fast ICA, INFOMAX ICA (Wan et al., 2008), ICA MERMAID (Marossero et al., 2003), and JADE ICA (Sameni et al., 2006) which were used for fECG morphological features were also tested on fPCG signals. A summary of the state-of-the-art signal processing algorithms used in fPCG analysis can be found in Adithya et al. (2017).

In line with the abovementioned background, we aim to apply a source separation algorithm that uses multi-lag covariance eigenvalue decomposition on fPCG signals because of its low computational complexity and suitability on non-independently mixed signals such as fPCG signals. Therefore, the aim of this study is to investigate if fHR signals extracted from fPCG and CTG are in good agreement.

## 2. Methods

### 2.1. Data collection and experimental setup

**Figure 1:**
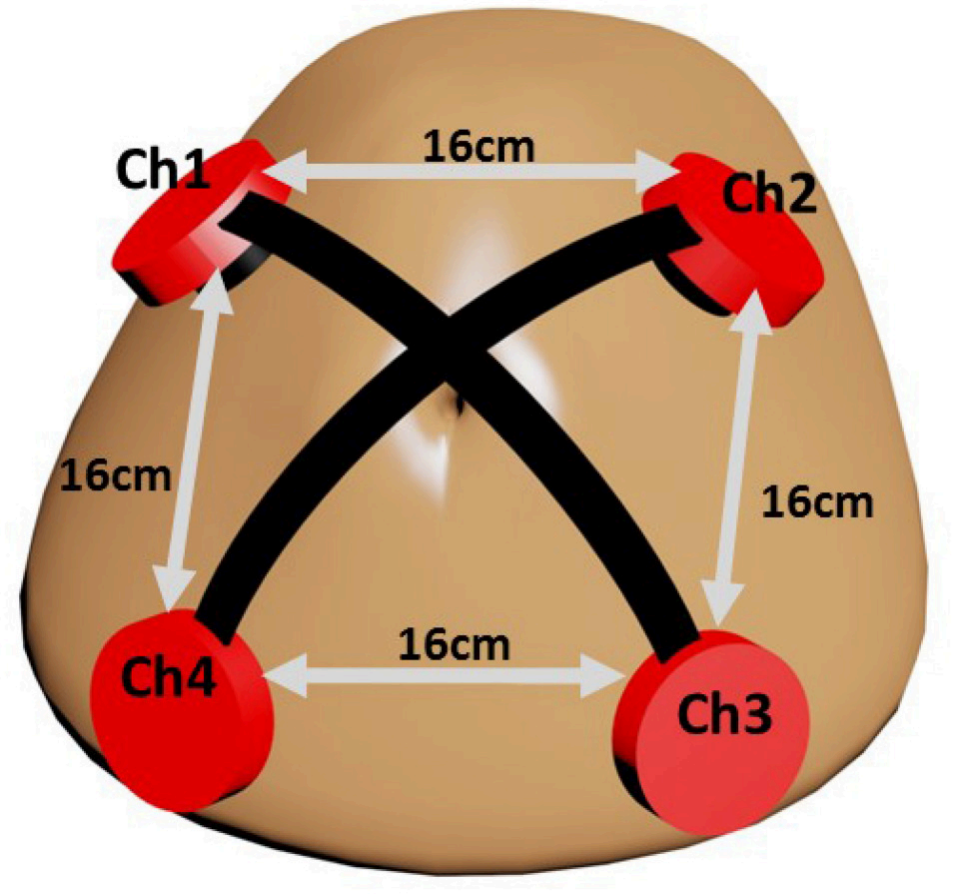
Piezoelectric vibrations sensors setup on the maternal abdomen.

The fact that fPCG acquisition is highly susceptible to noises paves the way to innovative hardware oriented methods for a less noisy acquisition. In this study, fPCG are recorded using vibration sensors (cost $1 each) embedded in high definition 3D printed plastic harnesses. Each harness holds a ceramic piezo vibration sensor (35 mm diameter) on the maternal abdomen with rubber made cushion to minimize the shear noise. The 3D printed harness is designed with precise parameters that rigidly mount the piezo sensor. The sketch in Figure 1 shows the setup of the sensors, where each sensor picks fPCG signals through a coaxial cable having very high insulating resistance. Power lab data acquisition system by ADinstrument (www.adinstruments.com) was used to record the abdominal phonograms at a sampling frequency of *f*_*s*_ = 1, 000Hz. In order to validate the extracted fHR, 14 simultaneous CTG recordings were collected by Monica wireless CTG AN24 (www.monicahealthcare.com) and, 6 by Phillips Avalon FM300 CTG (www.usa.philips.com) devices for 20 subjects in total.

**Figure 2:**
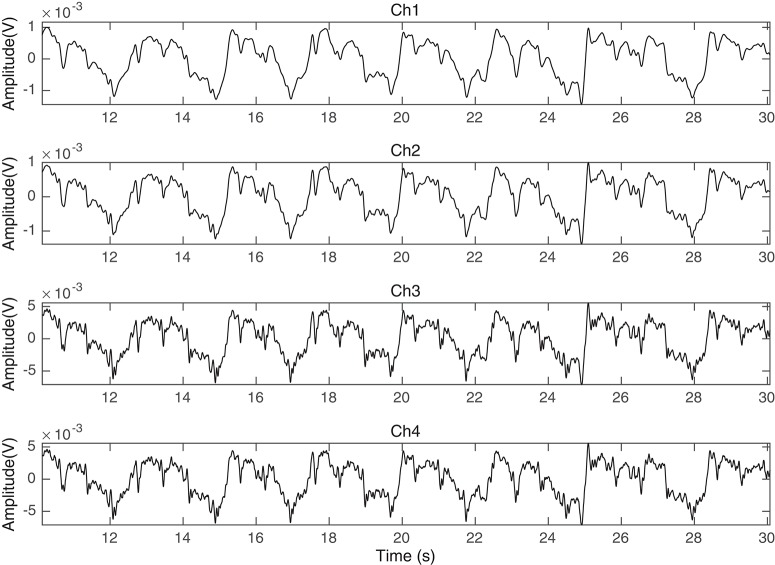
Ch1, Ch2, Ch3, and Ch4 of the fPCG recorded data in time (seconds) using the vibration sensors of Figure 1.

The prototype was tested on 20 pregnant mothers in Al-Ain Hospital, United Arab Emirates. Al Ain District Ethics Committee approved this study (ref: AAHEC-09-14-013) and informed signed consent forms were obtained from all pregnant volunteers. Figure 2 shows an example of recorded raw fPCG signal over time (seconds), which contains clear maternal breathing patterns (exhaling and inhaling) and fHS, as well, modulated, however, by the maternal breathing patterns, setting the challenge for efficient source separation. The total weight of the prototype (four channel sensors with harness) is 200 g and it was comfortable to wear which was confirmed by end of measurement survey questionnaire.

### 2.2. Proposed source separation technique

The special problem of extracting sources from a set of linear mixtures without any knowledge of the mixing channel has been widely studied by assuming different sources statistical conditions (Parra and Sajda, 2003; Naik and Wang, 2014). In its simplest form, it can be expressed as the problem of identifying the factorization of *P*-dimensional observations **x** into a mixing channel **A** and *Q*-dimensional sources **s**.

Let **x^o^**[*n*] be a multichannel discrete-time signal resulting from the synchronous sampling of *P* continuous-time signals (channels), where *n* denotes discrete time. The multichannel signal **x^o^**[*n*] corresponds to the linear combination of *Q* unknown source signals **s**[*n*] according to

(1) $$x^{o}[n]=As[n],$$

where **A** is the *P* × *Q* mixing matrix. The observation signals in **x^o^**[*n*] are given in row format (as well as the source signals in **s**[*n*]), such that $x_{i}^{o}\left[n\right]$ denotes the *i*th channel in **x^o^**[*n*]. The problem we deal with is the recovery of the sources **s**[*n*] from the observations **x^o^**[*n*], without any knowledge of the mixing matrix **A**.

In our particular problem, the observation signals **x^o^**[*n*] correspond to the synchronous reading of *P* = 4 vibration sensors. Given the sensor arrangement (Figure 1), placed 16 cm apart from each other, a distance much smaller than the minimum wavelength (λ = 3m in liquid for the specific *f*_*s*_), each source signal *s*_*i*_[*n*] arrives to every sensor with a different intensity, usually based on how close the sensor is to its respective physical source. We assume **s**[*n*] to be related to the mother's breathing, mother's heart, fetal heart, and noise, hence *Q* ≲ *P*. In consequence, the mixing matrix **A** is assumed to be of rank *P*, but not necessarily well-conditioned.

Let **e** be a diagonal control matrix to disable bad channels of *M* × *P* multiplied by **x^o^**[*n*], where *M* ≤ 4 is the number of strongly correlated channels, i.e.,

(2) $$x[n]=ex^{o}[n].$$

We define the observation cross-covariance matrix as

(3) $$X[n]=x[m]x^{T}\;[m+n],$$

where for such operation a *N*-point window is involved, that is, the *i*th row and *j*th column of matrix **X**[*n*] is obtained as follows

(4) $$X_{ij}[n]=\sum_{m=0}^{N-1}x_{i}[m]x_{j}[m+n].$$

Based on the mixing scenario (1), we can express matrix (3) in terms of the mixing matrix **A** as

(5) $$X[n]=As[m]s^{T}[m+n]A^{T}=AS[n]A^{T},$$

where **S**[*n*] is the source cross-covariance (unknown) matrix.

We are interested in a demixing matrix **W**^*T*^ such that **W**^*T*^**A** = **I** or **A**^*T*^**W** = **I**. This would separate the mixed signals as,

(6) $$s[n]=W^{T}x[n].$$

The expression in Equation (5) aims to provide general cross statistics between shifted versions of the collected sensors readings. Using Equation (3), we can refer to **X**[0] as the zeroth lag covariance matrix of the sensors recordings, while **X**[1] as the 1st lag cross-covariance matrix and **X**[*n*] as the *n*th lag cross-covariance matrix. These matrices will be used to devise an algorithm to recover the original components of a specific set of mixtures of data. In particular, starting from the zeroth lag it can be written

(7) $$\begin{array}{l} for\;n=0,X[0]=AS[0]A^{T}. \end{array}$$

In Equation (7), the sensors recordings zeroth lag covariance matrix **X**[0] is written in terms of the sources zeroth lag covariance matrix **S**[0] and the mixing matrix **A**. By multiplying each right side of Equation (7) by **W** it would result in

(8) $$\begin{array}{l} X[0]W=AS[0]. \end{array}$$

This process is repeated for *n* = 1, 2…*k* as shown below

(9) $$forn=1,X[1]=AS[1]A^{T},$$

(10) $$for\;n=2,X[2]=AS[2]A^{T},$$

(11) $$for\;n=k,X[k]=AS[k]A^{T}.$$

Blind source separation utilizing only the first lag covariance matrix **X**[1] has been shown and discussed in Parra and Sajda (2003), Weinstein et al. (1993), Parra and Spence (2000). Belouchrani et al. (1997) introduced a source separation technique utilizing the time coherence of the source signals. This relies only on stationary second-order statistics that are based on a joint diagonalization of a set of covariance matrices. In our mixing scenario, we assume that the sources are non-white, stationary and decorrelated. The aim here is to provide a simplified understanding of the stationary assumption by studying sums of multi-lag cross covariance up to *k*th-lag sample in a form of eigenvalue decomposition problem. As Equations (9–11) share the same mixing matrix **A**, let the *k*th sample be the discrete time lag over a very short period of time; the sum of the *n*th lag covariance matrices up to *k* is given by

(12) $$\begin{array}{l} \sum_{n=1}^{k}X[n]=A(\sum_{n=1}^{k}S[n])A^{T}. \end{array}$$

We multiply each right side of Equation (12) by a desired demixing matrix **W** to result in the following

(13) $$\begin{array}{l} (\sum_{n=1}^{k}X[n])W=A\sum_{n=1}^{k}S[n], \end{array}$$

and the original mixing matrix **A** can be written as,

(14) $$\left(\sum_{n=1}^{k}X[n]\right)W{\left(\sum_{n=1}^{k}S[n]\right)}^{-1}=A.$$

The goal here is to find the minimum sum to *k*th lag covariance matrix that constructs an eigenvalue decomposition problem to best approximate the demixing matrix **W**^*T*^. With the substitution of the original mixing matrix **A** derived in Equation (14) in the zeroth covariance form derived in Equation (8) we get

(15) $$X[0]W=\left(\sum_{n=1}^{k}X[n]\right)W{\left(\sum_{n=1}^{k}S[n]\right)}^{-1}S[0].$$

The above expression can be further written as

(16) $$\begin{array}{l} X[0]W=(\sum_{n=1}^{k}X[n])W\Lambda , \end{array}$$

where $\Lambda ={\left(\overset{k}{\underset{n=1}{\sum }}\text{S}\left[n\right]\right)}^{-1}\text{S}\left[0\right]$ is a diagonal matrix and **S**[0], **S**[1] …**S**[*k*] have the same diagonalization property over a short period of time.

For simplicity, we want to produces a diagonal matrix **D**=Λ of generalized eigenvalues and a full matrix **V**=**W** whose columns are the corresponding eigenvectors where **A**=**X**[0] and **B** in this case is nothing but $\overset{k}{\underset{n=1}{\sum }}\text{X}\left[n\right]$ so that the problem is in the form of an eigenvalue decomposition problem:

(17) $$\begin{array}{l} AV=BVD. \end{array}$$

The idea in Equation (16) is that the full matrix **W** is nothing but the eigenvectors matrix constructed not only from the covariance matrix **X**[0], but also from the sum of the minimum *k*th lag cross covariance matrix $\overset{k}{\underset{n=1}{\sum }}\text{X}\left[n\right]$. Because the sources are non-white, this method is described as using second order statistics in the form of cross-covariance for different time lags. In this way, the issue is transfered to the correlation maximization problem.

As previously discussed and shown in Equation (2), **e** matrix disables any bad channels or dismiss any operating window if they are not sufficiently correlated. A linear set of channels tend to have high degree of correlation because the independent components are available in every channel but with different weights. Let *C*(*i, j*) be the zeroth lag of a normalized covariance matrix between the *i*th and the *j*th channels; the correlation coefficient *R*(*i, j*) is, then, defined as

(18) $$R(i,j)=\frac{C(i,j)}{\sqrt{C(i,i)C(j,j)}},$$

where a matrix **R** written in a form of table. Table 1 gives the degree of correlation between all the channels. Performing such practice would give us valuable information on the channels similarities. The goal here is to extract a binary control matrix **e** to be multiplied by the raw collected signals **x^o^**[*n*] and disable any noisy (less correlated channels).

**Table 1:** Distinguishing good channels from bad channels.

	**Ch1**	**Ch2**	**Ch3**	**Ch4**	**Enable vector(*e*)**	**Decision**
**Ch1**	1	*R(1,2)*	*R(1,3)*	*R(1,4)*	*R(1,2) OR R(1,3) OR R(1,4)*	If output is logic 1
**Ch2**	*R(2,1)*	1	*R(2,3)*	*R(2,4)*	*R(1,2) OR R(2,3) OR R(2,4)*	enable the channel.
**Ch3**	*R(3,1)*	*R(3,2)*	1	*R(3,4)*	*R(1,3) OR R(2,3) OR R(3,4)*	If output is logic 0
**Ch4**	*R(4,1)*	*R(4,2)*	*R(4,3)*	1	*R(1,4) OR R(2,4) OR R(3,4)*	disable the channel.

*The black cells indicate mirrored version of the upper triangle covariance values; hence discarded*.

The upper triangle in Table 1 is converted to binary, based on a threshold value (initially set as 0.3); hence, any |*R*(*i, j*)| < 0.3 is set to 0 while those ≥ 0.3 are set to 1. Such practice would disable channels that are less correlated and would improve the blind source separation results. The control matrix **e** can be extracted from the logic *OR* gate of the *R*(*i, j*) values of the current channel with the remaining channels. The control matrix **e** is constructed as

$$e=\left[\begin{array}{cccc} e(1) & 0 & 0 & 0\\ 0 & e(2) & 0 & 0\\ 0 & 0 & e(3) & 0\\ 0 & 0 & 0 & e(4) \end{array}\right]$$

where if any *e*(*i*) value is set to zero the raw containing it is removed. This method can disable noisy channels and dismiss all of the sensors if all of them are noisy. Table 1 summarizes the proposed process.

The algorithm described in this section gives a close approximation of the original mixing matrix. In other words, the estimated demixing matrix **W^T^** is able to recover the independent components, which are the maternal breathing, mHS and the desired fHS, respectively.

**Steps through application:**

1. Construct input matrix: **x**[*n*] = **ex^o^**[*n*].
2. Find **X**[0].
3. Find $\overset{k}{\underset{n=1}{\sum }}\text{X}\left[n\right]$, up to predefined *k*th-lag sample.
4. Construct the eigenvalue decomposition problem: [**W**,**D**] = eig(**X**[0], $\overset{k}{\underset{n=1}{\sum }}\text{X}\left[n\right]$), where Λ=**D**.
5. Find the sources: **s**[*n*] = **W**^*T*^**x**[*n*].

### 2.3. Creating a synthetically mixed signal

**Figure 3:**
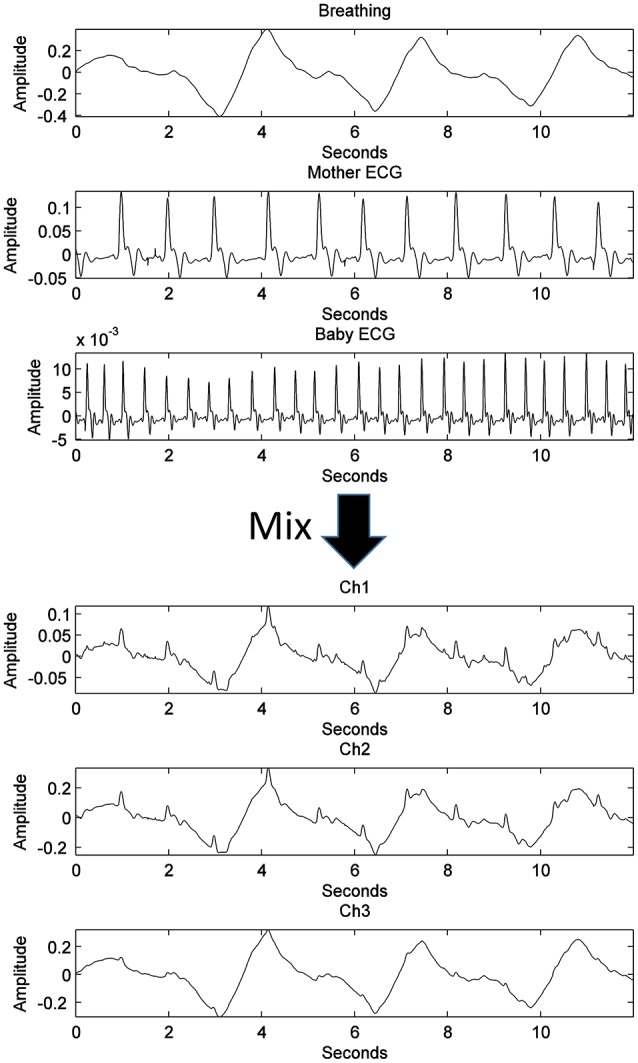
The process of generating three synthetic channels containing three mixed sources through a single iteration, randomly generated, well-conditioned matrix.

An actual maternal breathing pattern (recorded through vibration sensor), fetal ECG (fECG), and maternal ECG (mECG) signals were recorded to create synthetic channels. Because of the nature of the abdominal fading coefficients, the abdominal channel between the sources and the sensors are in continuous change due to mainly lung inhalation and exhalation and accordingly, its effect on the medium. In an attempt to mimic this scenario, 50 iterations of different positive normally distributed pseudorandom mixing matrices are used to mix the original sources as seen in Figure 3. The constructed mixing signals were then used to test the derived algorithm and to estimate the demixing matrix at each iteration with different *k*-values.

### 2.4. Applying the proposed algorithm on the sensors readings

**Figure 4:**
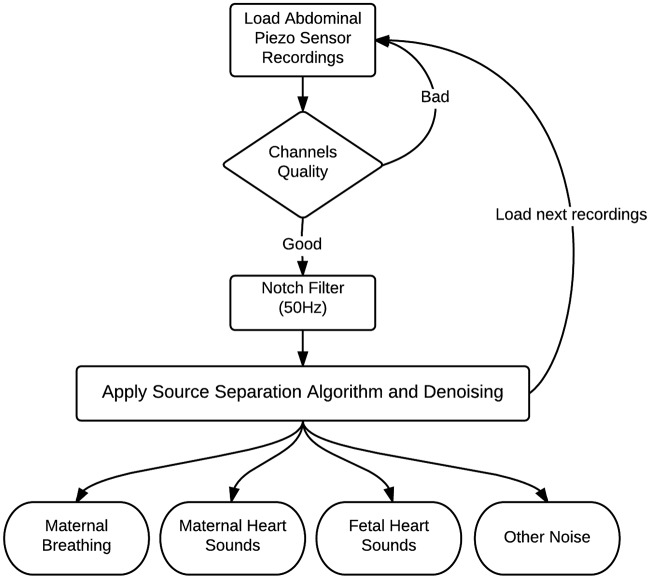
Block diagram of source separation process to separate the phonogram into maternal breathing, mHS, fHS, and other noise.

The source separation process for abdominal phonogram signals is described in Figure 4. The proposed method was tested on abdominal phonogram signals from 20 pregnant women; their anthropometric characteristics are listed in Table 2. Initially, the electric hum of 50 Hz was removed, using a notch filter with quality factor of 60. Then, the proposed source separation technique (Section 2.2) was applied to the filtered data and the mHS and fHS were extracted. These were further denoised using a wavelet transform-based stationary-non-stationary filter (WTST-NST) (Hadjileontiadis and Panas, 1997). The WTST-NST filter basically utilizes the multi-resolution wavelet decomposition with dynamic threshold values at each level to separate non-stationary signal components (noise) from stationary signal components (heart sounds). The peaks of the heart sounds were identified by finding the local maxima in a pre-specified peak-to-peak time distance of 0.3 s (assuming maximum fHR of 180 bpm). In this window, the data samples were compared to their neighboring values, where the peak of maximum energy was highlighted. If any element of data was larger than its neighbors, the element was considered as a local heart sound peak. The separated denoised fHS were then used to estimate the fHR for comparison with the same from the CTG machine.

**Table 2:** Summary of the tested subjects.

**Particpant ID**	**Maternal age (yr)**	**Weight (kg)**	**Height (m)**	**BMI (kg/m2)**	**HR (BPM)**	**Body temperature** (***c^o^***)	**Fetal movement**	**Gestational age (wks)**	**Maternal medical history details**	**CTG fHR (BPM)**	**PCG fHR (BPM)**
145	29	76.3	1.56	31.35	94	36.7	Unknown	40.6	No	135	133
152	30	61	1.59	24.13	99	36.7	Unknown	40.6	No	120	120
154	27	80	1.61	30.86	88	36.7	Unknown	41.2	No	134	135
158	31	75.5	1.55	31.43	93	36.7	Unknown	37.1	Bipolar Disorder	145	143
159	28	88.2	1.62	33.61	85	36.9	Yes	38	No	145	147
162	18	70.4	1.47	32.58	85	36.6	No	39	IUGR	130	130
168	39	56.7	1.5	25.02	90	36.6	Yes	37.1	Yes	140	141
169	40	89	1.64	33.9	82	37	Yes	34.1	Anemia, Hypertension, Hyperkalemia, bleeding p/v	120	123
182	26	63.5	1.56	26.09	90	36.8	Yes	36.1	GDM, Anemia	140	139
183	42	89.3	1.63	33.61	85	36.8	No	40.3	Anemia, Placenta previa, Vitamin D deficiency, Obesity	140	137
184	28	98	1.7	33.91	100	36.7	Yes	40	Acute lymphadenitis Anemia, Heartburn	132	132
185	32	85.6	1.65	31.44	85	36.9	Yes	35	GDM	140	142
187	28	84.5	1.61	32.06	81	37.1	Yes	40	No	125	125
188	32	81.4	1.61	31.04	90	36.7	No	34.6	Anemia	140	140
189	20	99.7	1.69	34.91	96	36.7	Yes	40	No	145	144
195	36	67	1.54	28.25	95	36.7	Unknown	41	Epilepsy	138	140
191	29	86.2	1.53	36.82	81	36.7	Yes	40	No	130	129
224	27	69.5	1.59	27.49	90	36.8	No	38.2	GDM, Bone and joint disorders of back	120	122
225	32	76.7	1.66	27.83	102	36.7	Yes	41.1	No	135	133
229	23	64	1.7	21.8	89	36.9	Yes	38	Anemia	140	141

#### 2.4.1. Fetal heart rate assessment

Based on the American College of Obstetricians and Gynecologists, obstetric medical assessment are those used to assess the medical condition of a fetus. For such purpose, many fHR pattern definitions have been classified and agreed (ACO, 2009, 2010; Graham Gaylord Ashmead, 2011). In this study, the fHRs derived from both the fPCG and the CTG approaches were used to assess only the baseline fHR values of 20 pregnant women participated in the study.

## 3. Results and discussion

### 3.1. Simulation

**Figure 5:**
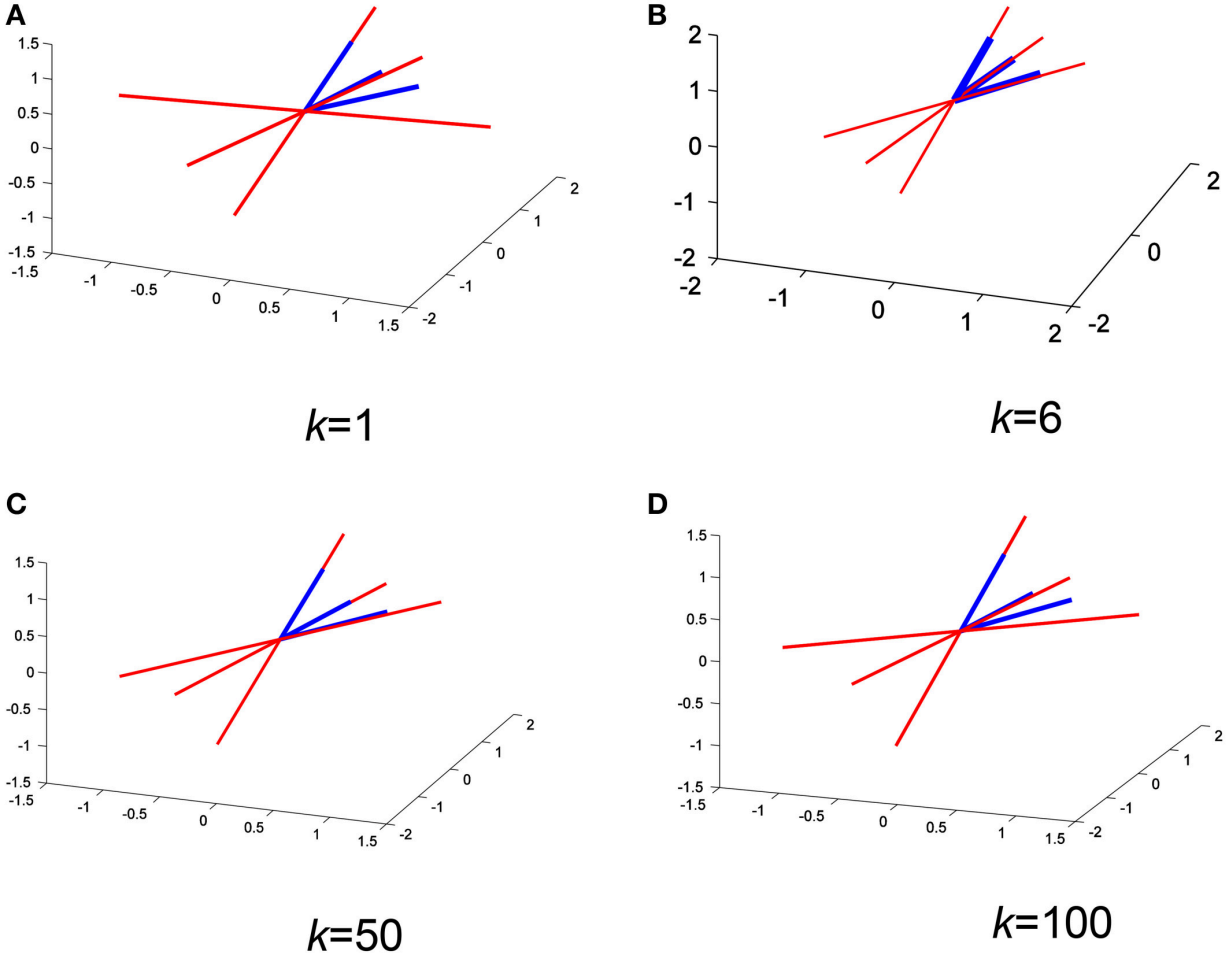
Overlapping the approximated mixing vectors (red) with the original ones (blue) for different *k* values **(A–D)** on a random iteration.

**Figure 6:**
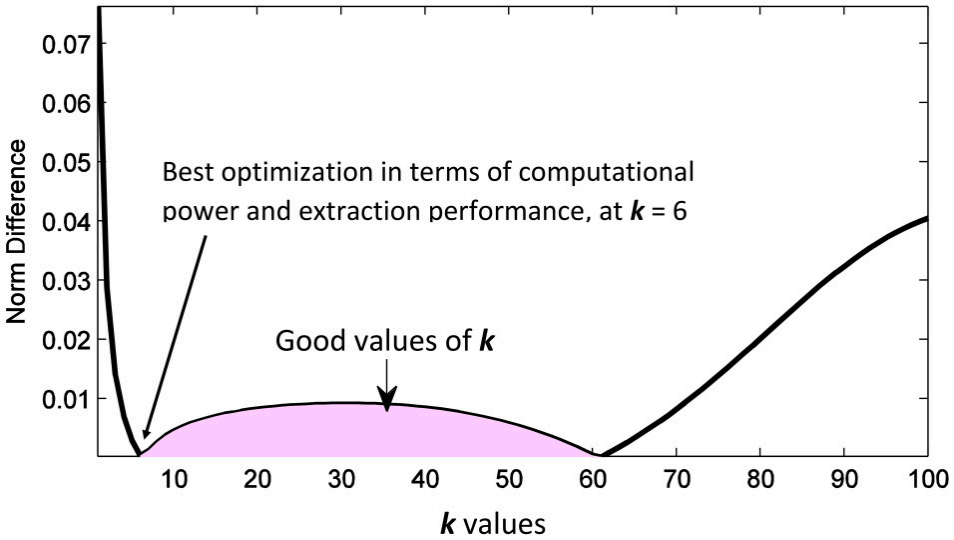
Random iteration: The abs 2-norm difference between the approximated mixing matrix and the original one, the shaded area indicates the good values of *k* to be used in the separation.

Figures 5A–D show a comparison between the approximated mixing vectors (plotted in red) using the expression derived in Equation (16) and the original mixing vectors (plotted in blue), for a full rank mixing matrix of 3 and for *k*= 1, 6, 50, and 100, respectively. The main purpose here is to overlap the vectors as much as possible or, in other words, minimize the 2-norm between the approximated mixing matrix and the original one. In addition, Figure 6 shows the abs 2-norm difference between the approximated mixing matrix **W** and the original one to indicate the suitable sum of the *k*th lag. The least 2-norm range occurs in the range from 6th lag (equivalent to 0.006 s) to a max of 60th lag (equivalent to 0.06 s) for the given *f*_*s*_ (1,000 Hz). The 6th lag sum gives the best approximation results, while minimizing the computational process. The 0.006–0.06 s gives the indication that in practical scenarios the time lagged cross-covariance matrices can not share across all the time lags the same **A**, because practically we can partially assume that the signal is stationary over a short period of time just as the 0.06 s. The sampling frequency here will not affect the ideology as generally we define its sufficient value to make sure that we detect the maximum needed frequency in our collected data. To be more specific, the advantage of using a 1,000 Hz sampling frequency appears when it comes to record the peaks of the detected fHS, a less sampling frequency would still collect the desired frequency component but would miss a peak point in fHS. It is very critical to obtain clear set of signals. The ECG data set that was collected to construct our synthetic model was recorded using AD instrument power lab four channel data acquisition system at a 1,000 Hz sampling rate, which produced smooth heart sounds. In industry, an ECG sampling interval of 1 ms is recommended to get accurate time domain measures. Accordingly, our PCG data were recorded at a 1,000 Hz, to acquire enough time domain measure. It is worth mentioning that a less sampling rate would result in a less number of time lags, but the time sample which happens to be at sample number 60 in Figure 6 would be missed in a less number of time lags. So, a less number of time lags due to a reduced sampling frequency would affect the resolution of Figure 6 and would not give us a clear picture of our kth-lag range.

**Figure 7:**
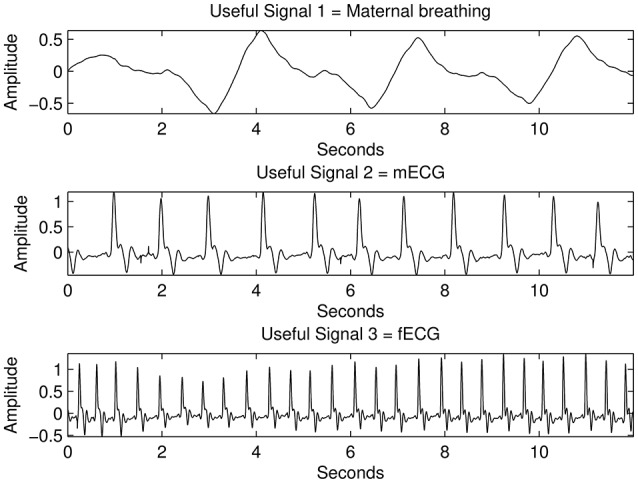
The results from the proposed source separation approach when applied to the simulated signals with mixed sources shown in Figure 3 for *k*=6.

Figure 7 shows the results of the proposed source separation approach when applied to the simulated data, using the 6th lag. When comparing the initial sources used for mixing (Figure 3) with the ones estimated in Figure 7, a clear source separation can be noticed, since the maternal breathing pattern, mHS and the fHS are correctly separated. In approximately all of the 50 iterations (program runs), the suitable *k*-value range was concluded the same.

### 3.2. Separating key fetal and maternal source signals

**Figure 8:**
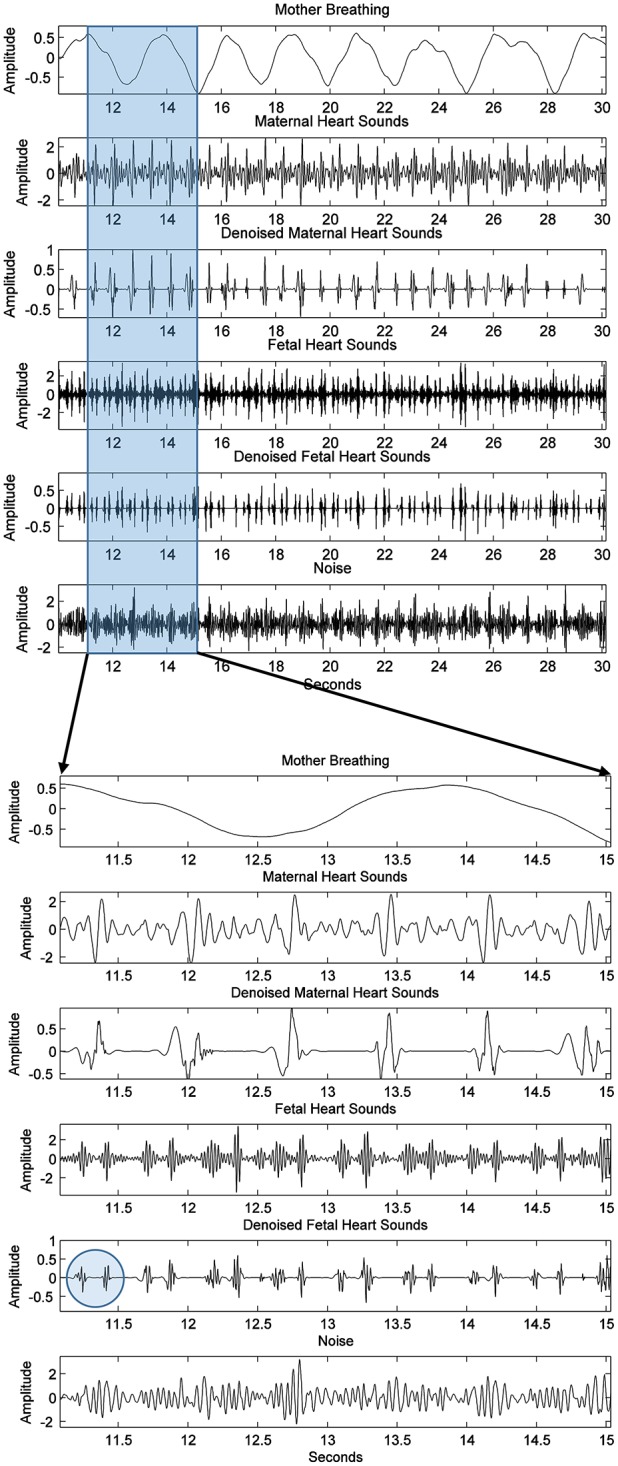
Subject ID 145: The results of extraction process of Figure 2 using the method mentioned in Figure 4, from the top to the bottom are the maternal breathing, mHS, fHS, and other noises. Two possible heart sounds (S1 and S2) of fetal heart are also seen and highlighted by a circle in the expanded view.

**Figure 9:**
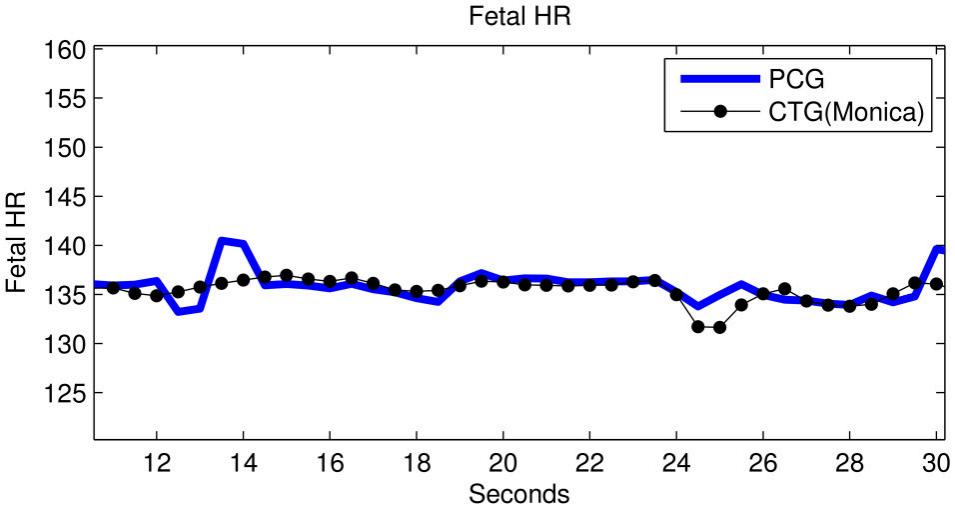
Subject ID 145: Comparison between fHR (bpm) obtained using fPCG vs. Monica wireless CTG AN24.

The raw data shown in Figure 2 of subject ID 145 were separated intro their source components, as seen in Figure 8, i.e., the maternal breathing, mHS, and fHS. The extracted fetal heart rates were then compared with the same obtained from Monica wireless CTG AN24 over the same time span, as shown in Figure 9. In general, the PCG-based fHR shows good similarity with the one obtained by Monica wireless CTG AN24, except from some sudden changes noticed in baseline (e.g., around 12th second in Figure 9). This is however expected, as Monica CTG AN24 uses a 3.75 s averaging window; hence, the original variation in fHR might have been lost due to this averaging process.

**Figure 10:**
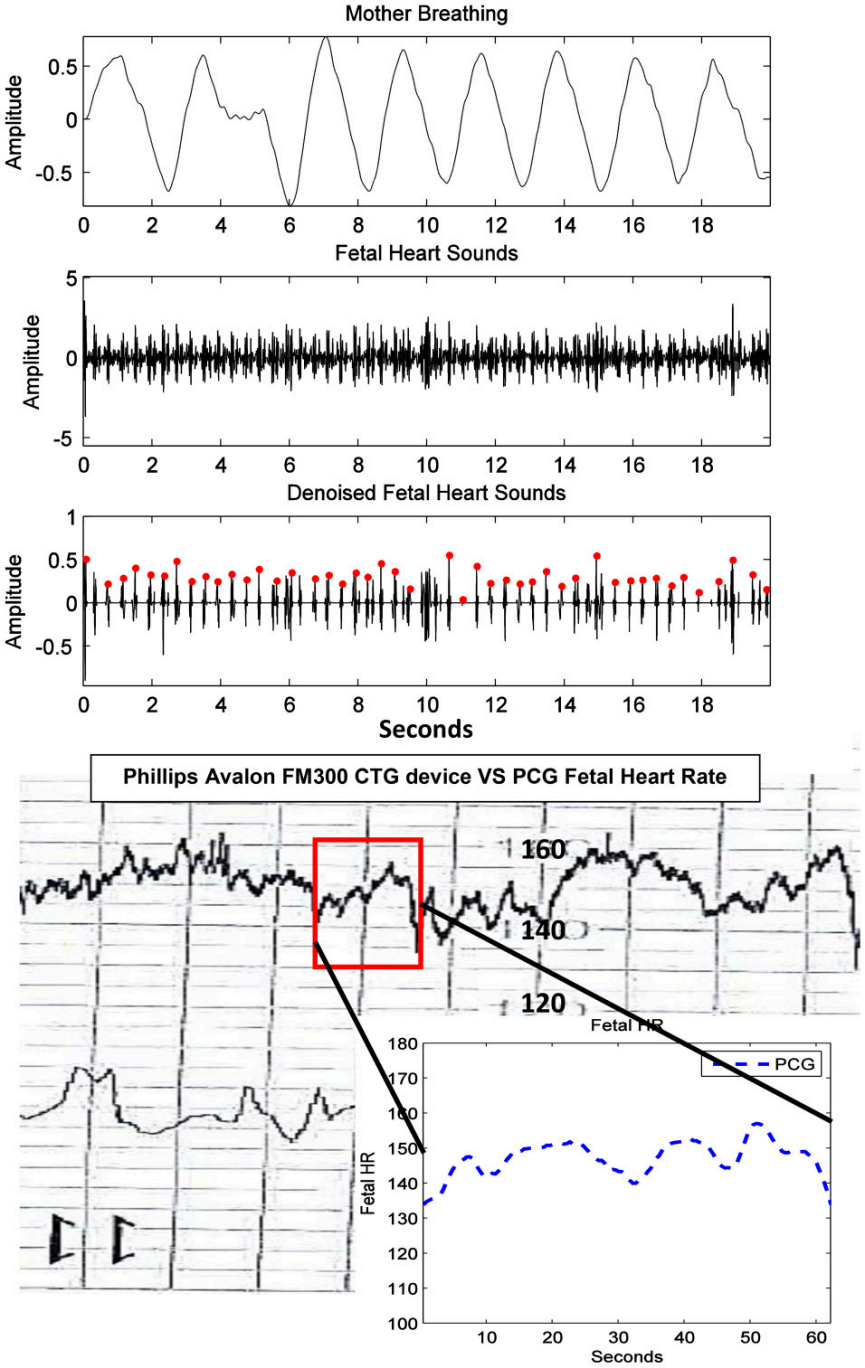
Subject ID 189: The results of extraction using the method mentioned in Figure 4, where the maternal breathing, fHS and (CTG vs. fPCG) fHR are shown.

**Figure 11:**
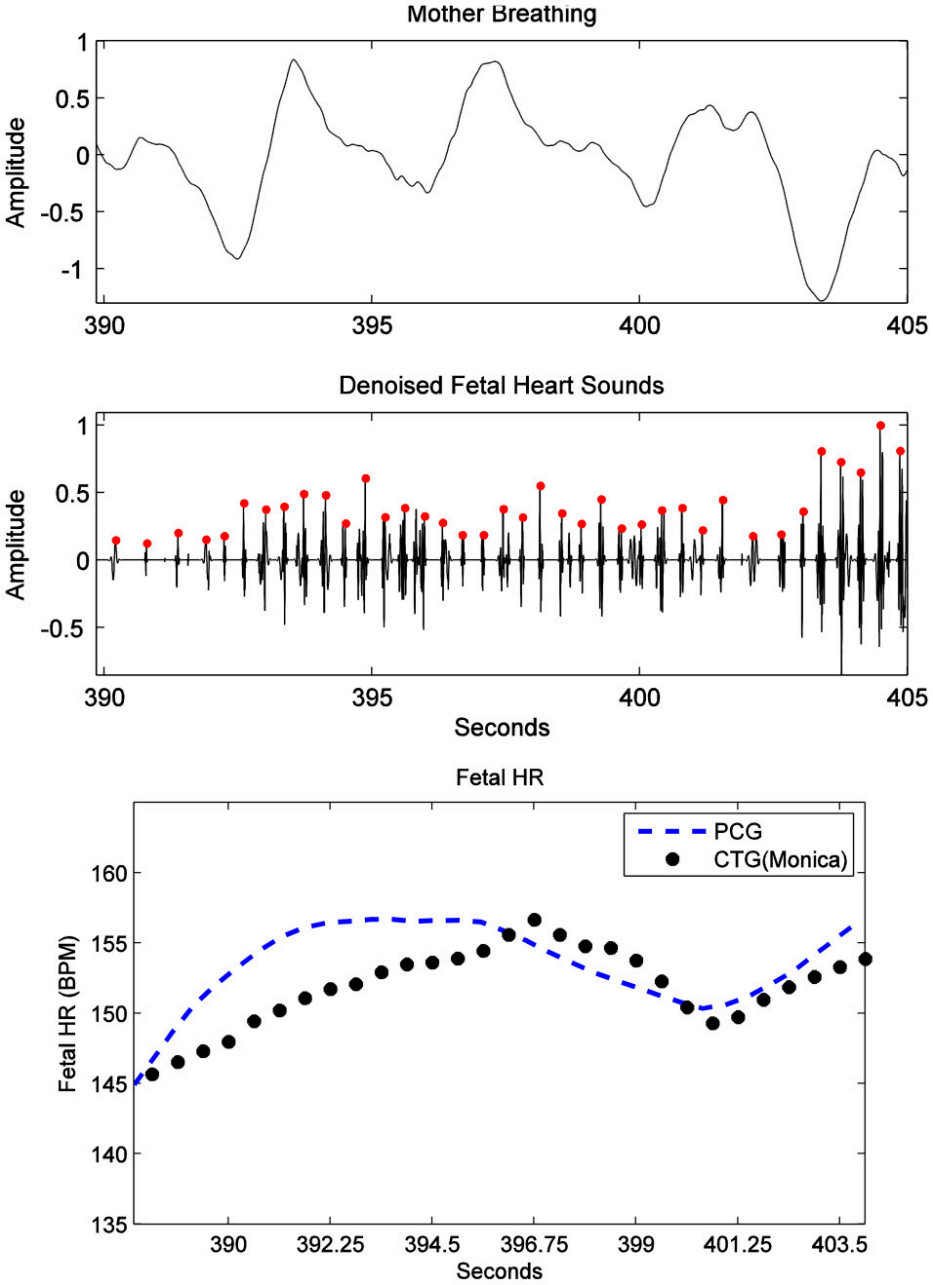
Subject ID 195: the results from the proposed source separation approach (Figure 4), where the maternal breathing, fHS and (CTG vs. fPCG) fHR are shown (top to bottom panels). Note that acceleration and deceleration periods have been picked by the PCG, in accordance to the ones picked by the Monica wireless CTG AN24, as shown in the bottom panel.

Furthermore, fHR extracted from fPCG were also compared with the same from the Phillips Avalon FM300 CTG device. In Figure 10, the fHR has been obtained from the same fHS panel but extended to 60 s (inset panel) to produce the same CTG portion highlighted within a square. The 60 s window is used, since the Phillips Avalon FM300 CTG device has a resolution of 60 s data/cm as the drawn 1 cm red square shows in Figure 10. The corresponding fPCG-based fHR (in the embedded subfigure of Figure 10 connected with the red square) shows good similarity with the same from the Philips device, as seen in the drawing pattern of the fHR, where the variability pattern is approximately similar. Acceleration and deceleration of the fHR are important signs for fetal well being. Figure 11 shows the denoised fHS detected of subject ID 195. Using fPCG-based monitoring, clear signs of acceleration and deceleration were noticed. The fPCG-based fHR were plotted and compared with the same from its Monica wireless CTG AN24 version, exhibiting a good matching in the acceleration/deceleration periods.

### 3.3. Overall comparative performance

**Figure 12:**
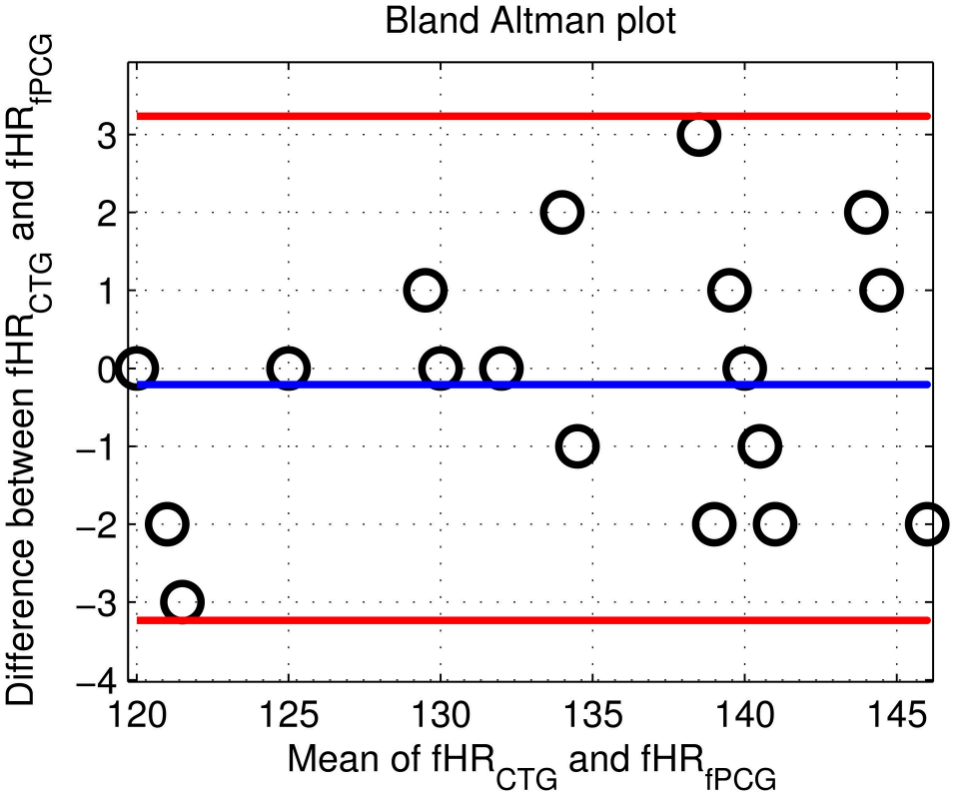
The Bland Altman plot of the fHR (fPCG vs. CTG) in the tested 20 subjects with mean = −0.21 BPM and ±2 *SD* = ±3 BPM.

**Figure 13:**
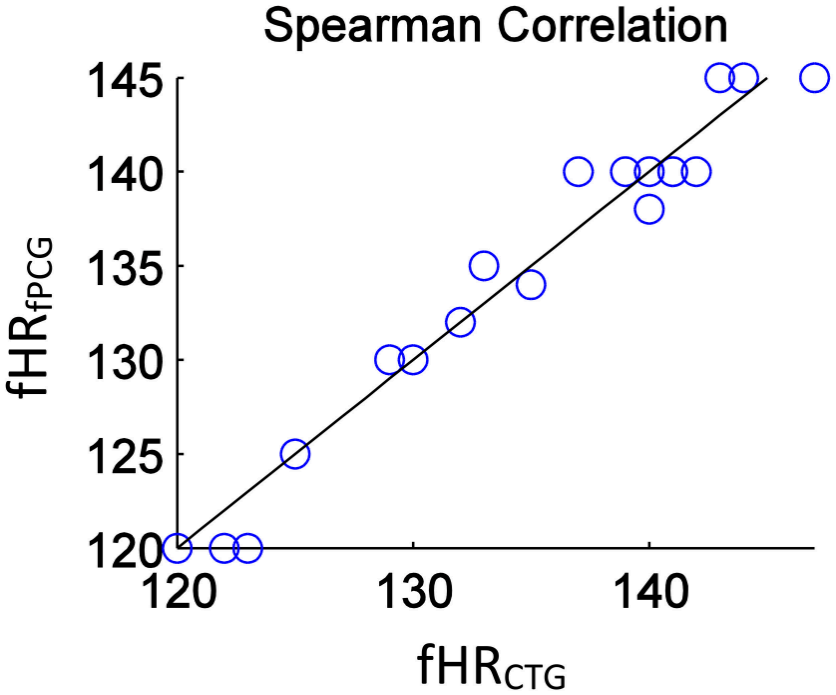
The estimated Spearman correlation coefficient of ρ = 0.95 (significance *p* < 0.001) yields a linear regression between **fHR_fPCG_** (_**bpm**_) and **fHR_CTG_** (_**bpm**_).

From an overall comparative perspective, Figure 12 shows the generated Bland-Altman plot of the mean fHR values (fPCG vs. CTG) in all 20 subjects, where Bland–Altman mean = −0.21 BPM and ±2 *SD* = ±3 BPM are identified. Moreover, Figure 13 depicts the Spearman correlation between the fHR from fPCG and fECG, respectively, exhibiting a ρ value of 0.95 (significance level of *p* < 0.001). Apparently, these results confirm that the fPCG-based fHR is comparable to those obtained by Monica wireless CTG AN24 and by Phillips Avalon FM300 CTG device. Such sensors setup and signal processing algorithm is of low cost and can be run on any PC and it is safe for long term monitoring. There is be no need for skilled operator to be able to operate the system, as the sensors harness can easily be placed on the abdomen, which could open up the possibility for home monitoring of fetal well-being.

The acceptable matches of mean fHR values and fHR baseline between the PCG and CTG data, verified by Bland–Altman and the Spearman correlation plots, support the application of PCG-based obstetric medical assessment rules on all the tested subjects. However, both PCG and CTG scan-based obstetric medical assessment suggested that the tested subjects had no urgent clinical issues in this study.

The novelty of this study includes the proof of concept that multi-channel low-cost vibration sensors harnessed in high definition 3D printed casings were able to reliably pick the fetal heart sounds. The control matrix **e** extracted from the OR logic of the zeroth lag covariance has been used to disable bad channels before applying any processing. Our proposed signal processing technique introduces the concept of summing multi-lag cross covariance matrices in an eigen value decomposition form. The multi-lag cross covariance sum is up to *k*th-lag sample. Depending on the final *k*th-lag cross covariance sum, the approximated demixing matrix performance changes, where we showed that multi-lag cross covariance sum up to *k*th-lag = 6–60 is reliable to separate fetal heart sounds from abdominal phonograms. In this acceptable range, choosing *k* = 6 gives the best optimization in terms of extraction performance and computational power as less sums will be computed. Besides, the multi-resolution wavelet decomposition with dynamic threshold values at each level to separate non-stationary signal components (noise) from stationary signal components (heart sounds) proved to be effective in denoising extracted noisy fetal heart sounds. The proposed method does not assume independent signals as this is a hardly met condition on abdominal surface due to different sources of noises. However, it looks at the best way to maximally decorrelate the sources. Due to the fact that it involves decomposition of the sources using extracted eigenvectors, puts it as a good candidate for fast processing specially in real-time processes. The length of the extracting vector equals the number of the maximally decorrelated channels, which imposes no storage limitation. The algorithm does not need any complex operations, while it only involves multiplications and additions which would not impose any heavy computation on the CPU. These advantages would qualify the algorithm to be run on any smart phone for domestic use.

The setup of our data acquisition system helped our recording environment to be as close to non-noisy as possible. The high definition 3D printed sensors holders harness robustly the vibration sensors on the abdomen that isolate them from possible unneeded external BSS context; such as, any speech signal. This along with our **e** control matrix narrow down the channels into maximally correlated ones. This helps our four sensors or less (depending on the **e** matrix) to receive the desired BSS context but with different linear combination due to the medium.

Possible unneeded abdominal BSS context; such as, muscle noise and baby kicks are not a big concern. These events don't happen all the time and can be tracked if a loss of fHS is witnessed. This is similar to the methodology used in the CTG scans.

However, further validations of PCG-based fHR on a variety of non-reassuring patterns of fHR, as seen in clinical scenarios, such as early decelerations, late decelerations, prolonged deceleration, recurrent, sinusoidal fHR, and variable decelerations are needed before the proposed technique could be used for a potential low cost antenatal care system. It is important to mention that we did not investigate how maternal weight such abdominal adiposity could influence the phonocardiogram signal quality in this study.

## 4. Conclusion

In this study, four channel low-cost vibration sensors in a square configuration harness were shown to capture fetal phonogram signals that were separated into source signals, such as fetal and maternal heart sounds and maternal breathing, by using a multi-lag cross covariance matrix-based eigenvalue decomposition technique. The carefully designed sensors casings by using HD 3D printing technology allowed us to dampen the noises caused by sheer due to maternal movements or external environmental disturbances. The validation results with clinically accepted standard CTG machines showed good agreements, which could open the future potential for such system to be used in clinical fetal monitoring. However, more validations on a variety of early, mid, and late gestational normal and abnormal cases are needed before it could be considered as a clinical fetal monitoring tool.

## Ethics statement

This study was carried out in accordance with the recommendations of Al Ain District Ethics Committee with written informed consent from all subjects. All subjects gave written informed consent in accordance with the Declaration of Helsinki. The protocol (AAHEC-09-14-013-Phonogram for Screening fetal well being) was approved by the Al Ain District Ethics Committee and United Arab Emirates University Ethics Committee (14/10).

## Author contributions

AK conceived the idea of low cost fetal phonogram device to extract fetal heart sounds. EI printed the sensors, derived and implemented the blind source separation, generated experimental results data, and drafted the Manuscript. LH generated MATLAB codes for noise cancellation method. SA and ZB carried out the experiments on pregnant mothers at Al Ain Hospital in UAE. AK and LH participated in the discussion and interpretation of the results. All authors read and approved the final manuscript.

### Conflict of interest statement

The authors declare that the research was conducted in the absence of any commercial or financial relationships that could be construed as a potential conflict of interest. The reviewer LE and handling Editor declared their shared affiliation.
